# Prenatal fruit juice exposure enhances memory consolidation in male post-weanling Sprague-Dawley rats

**DOI:** 10.1371/journal.pone.0227938

**Published:** 2020-01-28

**Authors:** Rachel Ward-Flanagan, Claire Scavuzzo, Piush J. Mandhane, Francois V. Bolduc, Clayton T. Dickson

**Affiliations:** 1 Neuroscience and Mental Health Institute, University of Alberta, Edmonton, AB, Canada; 2 Department of Psychology, University of Alberta, Edmonton, AB, Canada; 3 Department of Pediatrics, University of Alberta, Edmonton, AB, Canada; 4 Department of Medical Genetics, University of Alberta, Edmonton, AB, Canada; 5 Department of Physiology, University of Alberta, Edmonton, AB, Canada; Chiba Daigaku, JAPAN

## Abstract

**Objectives:**

Nutritional intake during gestation is known to impact health outcomes for progeny. Correlational evidence in humans suggests that increased fruit consumption of pregnant mothers enhances infant cognitive development. Moreover, wild-type *Drosophila* supplemented with a combination of orange and tomato juice showed robust enhancements in performance on an associative olfactory memory task. The current study aimed to experimentally test the effects of prenatal fruit juice exposure in a non-human, mammalian model of learning and memory.

**Methods:**

Across three separate birth cohorts, pregnant rats were given access to diluted tomato and orange juice (N = 2 per cohort), with control rats (N = 2 per cohort) receiving only water, in addition to standard rodent chow, throughout the duration of gestation, ending at parturition. Following weaning, male offspring were tested for learning and memory in a spatial version of the circular water maze and an auditory-cued fear-conditioning task.

**Results:**

All pregnant rats increased fluid and food intake over the gestational period. Fruit juice-fed pregnant rats had increased fluid intake compared to control pregnant rats. When testing progeny, there were no effects of prenatal fruit juice on spatial learning, while it appeared to impair learning in fear conditioning relative to controls. However, we measured significant enhancements in both spatial memory and conditioned fear memory in the prenatal fruit-juice group compared to controls. Measures of vigilance, in response to the conditioned cue, were increased in prenatal fruit rats compared to controls, suggesting less generalized, and more adaptive, anxiety behaviours.

**Discussion:**

Our results corroborate the human and *Drosophila* findings of prenatal fruit effects on behaviour, specifically that prenatal fruit juice exposure may be beneficial for early-life memory consolidation in rats.

## Introduction

A healthy diet during gestation is an imperative factor for proper neural formation and subsequently brain development [1]. For instance, prenatal folic acid supplementation has significantly reduced the incidence of neural tube defects in humans [2]. Conversely, dysregulation in carbohydrate metabolism can lead to functional and even structural defects in progeny. This has been illustrated by studies showing that increased maternal intake of sugar-sweetened beverages during pregnancy subsequently led to decreases in verbal intelligence in infants [3] and that holoprosencephaly is associated with gestational diabetes [4]. A recent meta-analysis assessing the impact of prenatal diet on infant cognitive outcomes found a significant effect of healthy maternal diet on infant cognition [5]. However, to translate into clinically actionable information, the specific prenatal dietary factors that could theoretically improve cognitive function, in the absence of nutrient deficiency, need to be conclusively identified [6].

To that end, a large-scale birth cohort study (Canadian Healthy Infant Longitudinal Development: CHILD) tracked the prenatal nutritional consumption of pregnant mothers and systematically followed the cognitive outcomes of their infants [7]. A positive relationship between self-reported maternal fruit intake during pregnancy and cognitive scores on the Bayley Scale of Infant Development III of infants at 1-year of age was identified. Specifically, for every daily serving of prenatal fruit, neurodevelopment scores increased by 2.38 points with consumption of up to 7 servings and 5.1 points for every serving beyond that. Multivariate analysis confirmed that prenatal fruit consumption effects on offspring neurodevelopment scores were maintained when controlling for socioeconomic status, gestational age, maternal education, gestational diabetes, maternal healthy eating index, and maternal vitamin supplementation. The CHILD study conducted a systematic analysis of the exact gestational nutrient intake, including: dietary fiber, calcium, omega 3 fatty acid, and calorie count, none of which were predictive of infant cognitive outcomes. However, both increased maternal lycopene and fructose intake, were associated with higher cognitive scores in infants. Given the findings of the CHILD study, where gestational fruit intake correlated with improved infant cognitive development, a series of follow-up experiments in *Drosophila* attempted to experimentally evaluate prenatal exposure to tomato juice (high in lycopene) and orange juice (high in fructose) on associative memory [7].

Interestingly, wild-type *Drosophila*, supplemented prenatally (throughout larval and pupation stages) with a 30% fruit juice addition to standard media made with equal parts of orange (15% volume/total media volume) and tomato juice (15% volume/total media volume), showed significant enhancements in short-term and long-term memory. Importantly, the enhanced memory effect of prenatal fruit supplementation persisted when 1 week old flies, exposed to prenatal diet enrichment, were transferred to regular media in postnatal period and submitted to the same training protocol, suggesting long-lasting changes from the prenatal exposure. Yet, supplementation with *either* orange *or* tomato juice (30% volume/total media volume) alone, or supplementation with 3% sucrose did not produce significant memory enhancements. The beneficial effects of prenatal fruit supplementation on offspring cognition observed in both human and *Drosophila* studies, were exhibited in both male and female offspring, suggesting that the effects of prenatal fruit on cognition are likely independent of sex [7].

Our aim in the present experiment was to test for cognitive effects of prenatal fruit juice supplementation in a non-human, mammalian model. Pregnant rats were supplemented with an equal ratio of tomato and orange juice during the gestational period specifically, and this supplementation was withdrawn post-parturition. Male offspring were weaned at post-natal day 18, and assessed in both spatial and emotional learning and memory tasks. Our aim was to employ a developmental time point analogous to the first year measure used in the CHILD study and to further evaluate this beneficial effect on cognitive development, experimentally, in a mammalian model system.

## Methods

### Subjects

Sprague Dawley rats bred in the Sciences Animal Support Services facility at the University of Alberta were used in this study. All experimental procedures were approved by the Biological Sciences Animal Care and Use Committee of the University of Alberta, in accordance with the guidelines of the Canadian Council on Animal Care (CCAC). Animals were housed on a 12 hour light/dark cycle (lights on at 7am).

### Paradigm for the supplementation of gestational diet with fruit juice

Pregnant rat dams (n = 12) were used across three separate experimental runs, with 4 dams per experimental cohort. Dams were randomly assigned into experimental (gestational fruit juice supplementation) or control (water) groups. All groups received free daily access to 50 g of standard breeding colony rat chow: PicoLab Rodent Diet 5053 (LabDiet, Brentwood, MO, USA). We calculated the minimum beneficial gestational juice intake of dams in the experimental group to be on the order of 20–30 ml of fruit juice per day, based on data from the CHILD study. In the CHILD study, cognitive outcomes were markedly improved in infants born to women who ingested 7 or more servings of fruit per day. Given an average body weight of 62 kg for pregnant women in the study and a single serving of fruit being equivalent to 250 ml of fruit juice, we calculated that this would be equivalent to at least 28 ml/kg of pure juice (250 ml x 7 servings / 62 kg = 28 ml/kg). Therefore, dams in the experimental group (averaging below 500g body weight) received access to a daily cocktail of 40 ml equal parts tomato and orange juice (Heinz, North York, ON, Canada; Minute Maid, Toronto, ON, Canada) diluted to 80 ml to negate any preferential choice between water and juice, while the control dams received 80 ml of water daily. Food and fluid intake were measured every 24 hours throughout gestation to ensure dams were obtaining comparable amounts of food and liquid. Although the CHILD study did not detect associations in fruit consumption and gestational diabetes, they did note that gestational diabetes by itself interacted with fruit intake on infant cognitive scores [7]. Thus, to ensure the fruit juice protocol used in our study did not induce gestational diabetes, we measured levels of HbA1C one month following parturition. HbA1C levels were similar in both groups of fruit-juice fed and control rat dams.

### Paradigm for the Post-parturition diet

**Fig 2:**
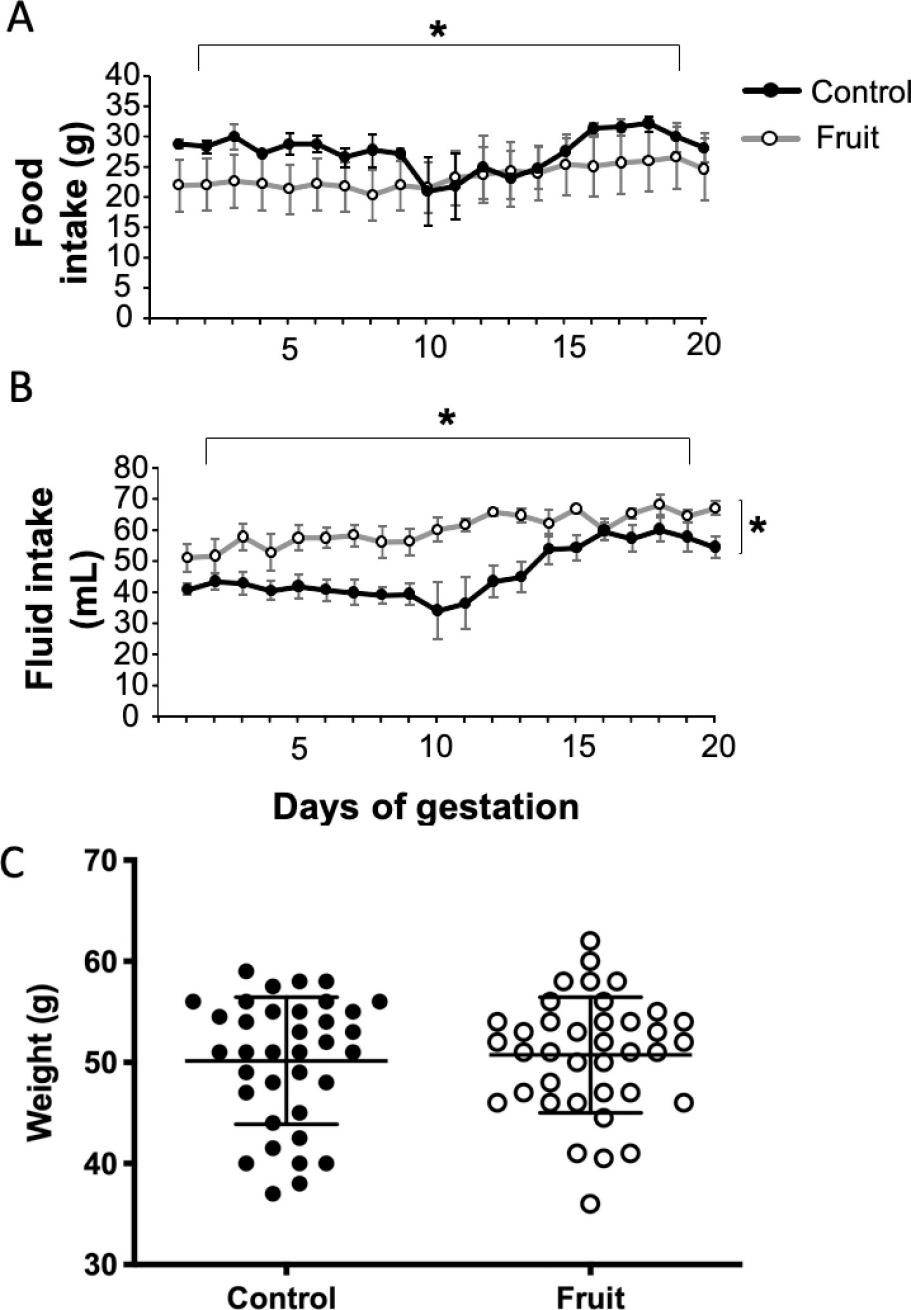
Food and fluid intake of pregnant dams throughout gestation, and weaning weights of offspring. A) Throughout gestation, there was an overall increase in chow intake in both fruit juice-fed and control rats. However, there was no effect of fruit juice exposure on chow intake. B) Throughout gestation, both fruit juice-fed and control rats increased fluid intake as the pregnancy progressed. However, fruit juice-fed rats had a significantly increased fluid intake over the pregnancy compared to control rats. C) At weaning (postnatal day 18) male offspring of control and fruit juice-fed rats had similar weaning weights. * = *P*<0.05. See results for exact p-values and statistical tests.

Post-parturition, access to fruit juice was withdrawn from the dams in the experimental group and all dams were returned to normal ad libitum access to food and water during the lactation period. Offspring were weaned 18 days postnatally, as behavioural testing and handling of rats required several days and we wanted to test the offspring at an age analogous to one year in humans, and prior to the onset of puberty. Although the weaning age of 18 days was younger than the conventional 21-day weaning, rat weanling weights were comparable to a 21-day-old rat (≥40 g) ([8]; see Fig 2C). Once weaned, offspring were weighed, housed with littermates, with no more than 4 siblings per cage, and given ad libitum access to food and water. A total of eighty-two male offspring were assessed on behavioural measures of cognition over three separate experimental cohorts (see Table 1). To ensure that all rats could be tested at the same circadian times and within the same developmental time points, litters were culled to a maximum of ten males. Due to infrastructure, circadian, and researcher time constraints following the weaning of a birth cohort (containing 4 litters per cohort), and the length of time required for each behavioural assay, only a single sex (i.e., males) were assayed behaviourally within each birth cohort.

**Table 1:** Number of male offspring assessed per litter over the 3 experimental cohorts.

	Cohort 1	Cohort 2	Cohort 3
Fruit-fed Dam 1	7	7	8
Fruit-fed Dam 2	7	5	9
Control Dam 1	10	5	8
Control Dam 2	9	7	0

A total of 82 rats were evaluated across 3 separate cohorts. Despite the loss of one litter in the last cohort (due to the dam consuming her young), there were no significant differences in litter sizes between fruit-fed and control dams (t_(10)_ = 0.43, *P* = 0.68).

Behavioural tests were always conducted in the order of circular water maze followed by fear conditioning. All rats began behavioural testing between P19—P21, whereupon progeny were trained on the circular water maze for four days, followed by a probe trial testing memory 24 hours later on the fifth day. This particular age was chosen as it is upon the developmental cusp for rats to learn the spatial navigation necessary to complete the circular water maze task [9]. Animals were then handled on four consecutive days, for five minutes a day, to habituate the animals to the experimenter and reduce fear arising from handling which could have increased freezing behaviour during the fear conditioning protocol. Following the four handling days, animals were then habituated to the shock and test chambers for 10 minutes each over 2 consecutive days to attenuate contextual fear. Fear conditioning training occurred on the following day, and testing occurred 24 hours later. All behavioural tests commenced at 9 am and concluded by 6 pm (lights on at 7 am). Testing for spatial memory (circular water maze probe trial) and fear conditioning occurred 8 days apart.

### Visuo-spatial memory testing with circular water maze

**Fig 1:**
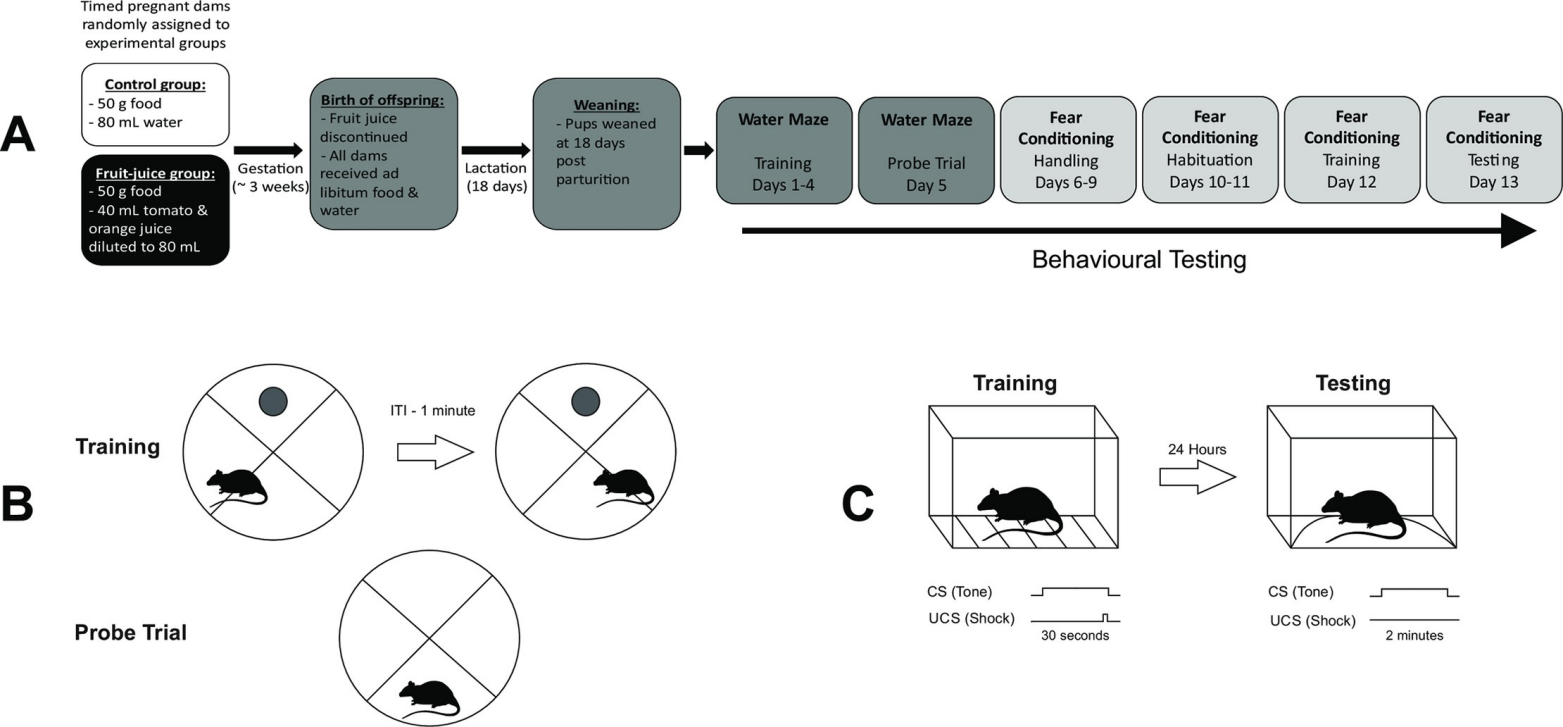
Experimental timeline and behavioural tasks. A) Timeline of the experimental experimental paradigm. Pregnant dams were randomly assigned into either the fruit juice exposed or control group. Following the birth of offspring, fruit juice was discontinued in the experimental group and all dams were switched to ad libitum food and water for the lactation period. Offspring were weaned at post-parturition day 18 (P18) and weighed. Behavioural testing of offspring began on P19 with the circular water maze followed by the auditory fear conditioning task, lasting 13 days total. B) The modified circular water maze task. Rats were trained over 4 days with 2 trials per day. Each trial was separated by a 1 min inter-trial interval. The platform location remained constant during training trials across days, and the start location of the rat was semi-randomized across all training trials. The probe trial was conducted on the 5^th^ day, in which the platform was removed from the maze, and rat was placed in a novel start location. C) Auditory-cued fear conditioning task. Animals were exposed to an auditory cue (conditioned stimulus: CS) for 30 seconds that co-terminated with a 1 second foot shock (unconditioned stimulus: UCS). This pairing was conducted twice with a one minute rest interval. The following day, rats were placed in a different chamber in a different room. Following a 2 minute delay, animals were played the same tone (CS) that had previous been paired with the shock (UCS). Freezing behavior was measured in 5-second epochs over the entire baseline and CS presentation periods.

Spatial learning and memory were assessed using a modified circular water maze paradigm, designed to be more difficult than other multi-trial iterations of the maze in order to eliminate any ceiling effects in performance ([10], see Fig 1A). The circular water maze (diameter 152 cm, depth 46 cm) was in a dimly lit (14 lux) room surrounded by extra maze cues. Subjects were given only 2 trials per day over 4 days, with an inter-trial interval of 1 minute, to learn the location of the hidden escape platform. For each learning trial, rats were lowered into the water by the experimenter into one of four semi-randomized quadrant locations, to ensure each location was not used more than once per day [11]. Subjects were then allowed to swim until the platform was located, or until 60 seconds had elapsed, at which point the subjects were guided to the platform by the experimenter if the platform had not yet been located by the subject. Once on the platform, subjects were allowed 10 seconds to assess extra-maze spatial cues before the end of the trial. The location of the platform remained the same across days in order to elicit a search strategy reliant upon memory for extra-maze spatial cues. Learning was defined by a decrease in latency to locate the platform over training trials.

On the fifth day, a probe trial was conducted wherein the platform was removed, and the subject was placed in a randomized quadrant and allowed search for the platform for 60 seconds. Assessment of memory strength for the platform location was evaluated based on the latency to locate the platform (in seconds), time spent in the target platform–quadrant versus non-target quadrants, the frequency of platform crosses, and whether the previous platform location was located in the first 30 seconds of the trial. All circular water maze trials were recorded and analyzed using EthoVisionXT video tracking software (Noldus, Wageninen, The Netherlands).

### Emotional memory testing using cued fear-conditioning

An auditory cued fear-conditioning task was employed to assess classical conditioning memory comparable to the odorant avoidance paradigm previously used in the *Drosophila* model of prenatal fruit supplementation [7]. Initially, subjects were handled for five minutes a day for four consecutive days to habituate to the experimenter. Subjects were then habituated to both the shock chamber (20.0 cm by 30.0 cm wide and 19.0 cm tall; composed of metal with an electrifiable grid floor), and the test chamber (20.0 cm by 30.0 cm wide and 26.0 cm tall; composed of colored Plexiglas, with one curved wall) over two days for 10 minutes each to reduce potential anxiety caused by a novel environment (see Fig 1B).

Prior to the conditioning trial, the shock chamber was cleaned with 70% ethanol for each subject, and the room lights were dimmed (to 2 lux). Once the task began, subjects were allowed to freely explore the chamber for two minutes to establish a within-subject pre-shock baseline of freezing. Following the two minute baseline, a speaker in the chamber played a 5 kHz tone for 30 seconds, which co-terminated with a 0.65 mA foot shock that lasted for 1 second. Foot shocks were administered by a scrambled output shock generator (HSCK1000, Lafayette Instruments, Lafayette, IN) through the grid floor. A second tone and shock pairing were presented 60 seconds following the first pairing in order to re-inforce the conditioned memory. The rat then remained in the chamber for an additional 60 seconds. Learning in this task was defined as an increase in the percent of time spent freezing comparing the last 30 seconds of baseline to the first 25 seconds of the tone playing, for both tone trials. Once the conditioning session ended the rat was placed in a holding cage until all rats completed the conditioning session, whereupon all rats were placed back in their respective home cages.

Following a 24 hour interval, subjects were tested for the strength of their memory of the tone-shock pairing in the test chamber in a different room with bright lighting (67 lux). Prior to the trial, the test chamber was cleaned with a 5% acetic acid solution to provide a novel scent from original training chamber. The test trial consisted of a two minute baseline period, followed by a two minute test period in which the 5 kHz tone was played continuously, with no shock administered. Assessment of memory for the conditioned fear task was evaluated by the percentage of time the animal spent freezing to the auditory cue compared to the percentage of time the animal spent freezing during baseline. Freezing was operationally defined as when rats were completely immobile (except for respiratory movements) while simultaneous maintaining an erect posture. All conditioning and test trials were filmed and scored by the experimenter in 5 second epochs in a binary fashion (freezing or not freezing). Learning was operationally defined as increased freezing to the cue + footshock pairing on the second trial of conditioning, whereas memory was defined as increased freezing to the auditory cue on the testing day (24 hours following conditioning).

### Statistical analysis

Due to the rats in our study being born across 3 different cohorts, the data presented herein is a combination of the raw data from all 3 cohorts. We additionally performed analyses that controlled for cohort effects by normalizing measures to control values within each cohort, which produced similar results when compared to the raw data. To normalize data, and percentage of control values, the mean of the control litters within each cohort was subtracted from the individual measures from each animal (x) and divided by the mean control (*μ*), and to get a measure of % of control values, multiplied by 100. As seen here: ($\frac{\left(x-\mu \right)}{\mu }$)*100. Unfortunately, there was not sufficient power within each cohort to make separate within and cross cohort comparisons. Thus, we report only the raw data, or permutations of the raw data, and have made our conclusions based on the overall effects observed when cohorts are combined and compared by treatment.

#### Food & Fluids

Ingestion of food and fluids of pregnant dams was measured daily throughout gestation. A 2-way repeated measures ANOVA assessed for changes in fluid and chow intake over the 3-week gestation period in fruit juice-fed versus control rat dams. Two-tailed, unpaired, Student’s *t*-tests were used to compare weaning weights of prenatal fruit versus control offspring. Student’s *t*-tests. Pearson r correlations were performed to determine relationships between fluid intake during pregnancy to memory scores in the water maze and fear conditioning tasks. To control for nutrient intake in the fruit-juice fluid, correlations were done within that treatment group specifically. Significance was set at α = 0.05 throughout.

#### Circular water maze task

For learning in the water maze, repeated measures 2-way ANOVAs assessed for changes in latency (seconds) to locate platform across training trials and interactions between treatments and time to learn. In the water maze, spatial learning was operationally defined as a decreased latency to locate the platform across test sessions. Spatial memory during the probe trial was operationally defined by four measures: an increased number of platform crosses, a decreased latency to locate the platform (both of which were compared across treatments using Two-tailed, unpaired, Student’s *t*-tests), the rat’s ability to locate the platform (which compared the proportion of control and fruit rats that did or did not locate the platform using a chi squared analysis), and increased time in the target quadrant (which compared the amount of time spent in target vs non target quadrants across treatments using a 2-way ANOVA).

#### Cued fear conditioning task

In order to assess changes in freezing behavior during the test trial, the proportion of time spent freezing over 30 second epochs in baseline and freezing periods was analyzed using a repeated measures 2-way ANOVA for treatment and freezing over time. To compare average freezing during baseline to average freezing during the tone period, proportion of time freezing was averaged over the whole baseline period (2 minutes) and compared to the average freezing to the tone over the whole cue period (2 minutes). A repeated measures 2-way ANOVA compared treatments and average freezing over baseline and tone periods.

Additionally, we controlled for baseline freezing within animals, because some rats froze and startled during baseline, indicating a higher likelihood of startling or freezing during testing. Emotional learning and memory was operationally defined as a bias in increased freezing to the auditory cue over baseline freezing. This bias was calculated by subtracting the average of baseline freezing from average freezing to the tone and dividing by the sum of baseline freezing and freezing to the tone. This was then multiplied by 100 to indicate the percentage of bias in freezing: (average proportion freezing to tone–average proportion freezing in baseline)/(average proportion freezing to tone + average proportion freezing in baseline) * 100. Bias to the tone percentages were then compared across treatment groups using two-tailed, unpaired, Student’s *t*-tests.

To assess vigilance in freezing behavior we compared freezing during the first 30 sec (tone 1) of the tone playing during the memory task to the last 30 seconds the tone is played (tone 4). A repeated measures 2-way ANOVA compared treatments and proportion of freezing over tone 1 vs tone 4 periods.

## Results

### Food, fluid intake and body weights

We assessed daily food and fluid intake across the period of gestation for differences across the two experimental conditions. A 2-way repeated measures ANOVA showed that chow intake significantly increased over time across all dams (F_(19,171)_ = 2.5; *P* = 0.0007; Fig 2A), but there was no effect of fruit juice exposure on overall chow intake (F_(1,9)_ = 0.02; *P* = 0.87) nor was there any interaction with gestation time and exposure to fruit juice (F_(19,171)_ = 1.3; *P* = 0.16) on chow intake. There was also an overall increase in fluid-intake over time (F_(19,171)_ = 7.78; *P*<0.0001; Fig 2B). In this case, there was a significant effect of exposure to fruit juice, such that fruit juice-fed rats drank more fluid overall compared to control rats (F_(1,9)_ = 13.52; *P*<0.0001). Also, there was a significant interaction of fruit juice exposure and gestational days on fluid ingested (F_(19,171)_ = 2.178; *P* = 0.0046). The experimental dams drank amounts of fruit juice that, based on body weight and extrapolations from the findings from the CHILD study [7], would be expected to yield cognitive benefits, with fluid intake of at least 40 ml of the diluted solution per day which would provide the minimum 20 ml of the actual fruit juice mix.

Despite the increase in fluid intake for the dams exposed to fruit juice supplemented diet, the post weaning weights of prenatal fruit juice versus control pups were not significantly different (Fig 2). There was no change in weaning weights from control when looking at combined raw weights (t_36_ = 0.41; *P* = 0.68; Fig 2C).

### *Visuo-spatial learning in circular water maze*: Prenatal fruit juice treatment does not have an effect on learning

**Fig 3:**
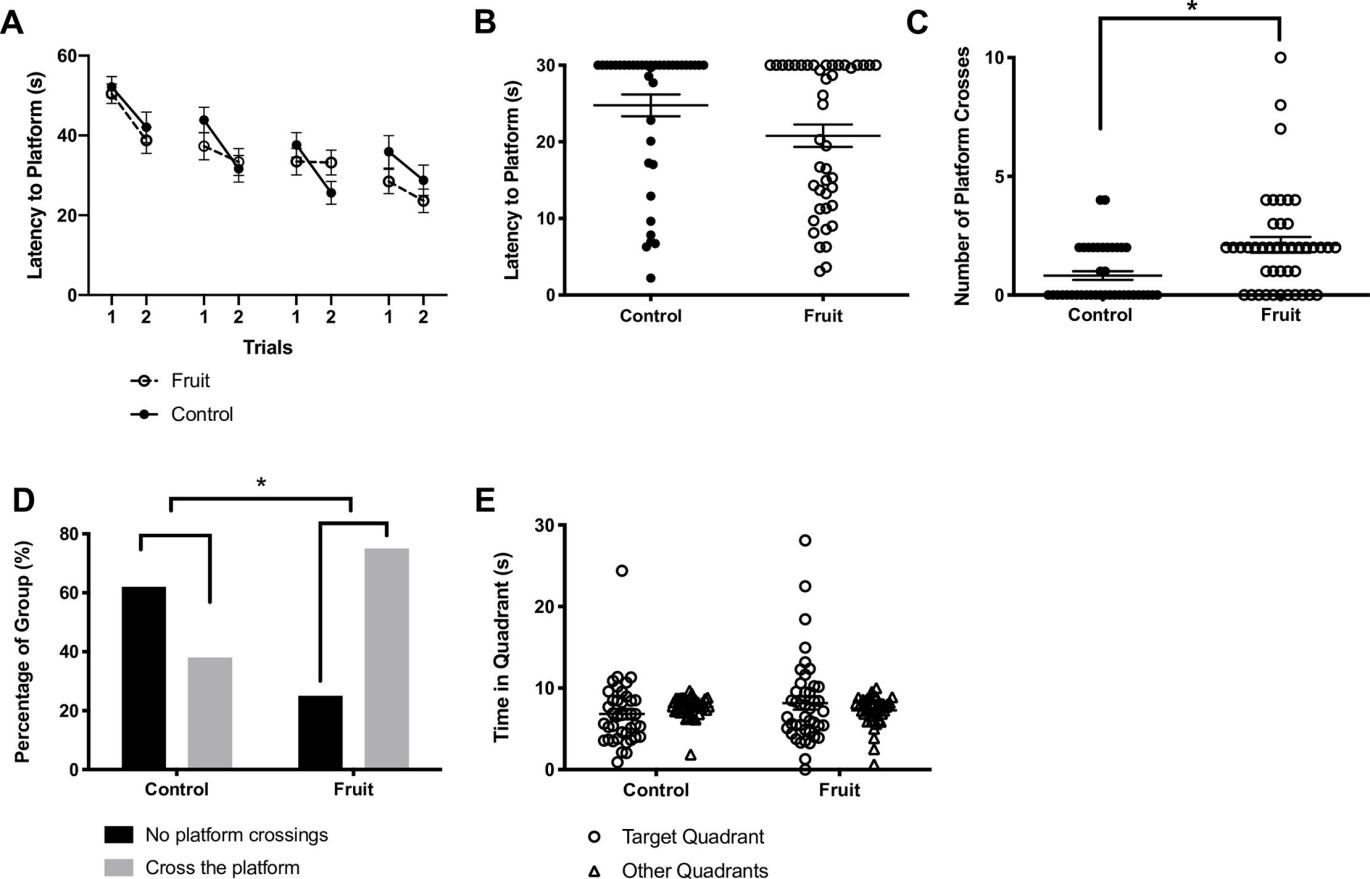
Spatial learning and memory in the water maze. A) Although learning occurred in all rats, there were no significant treatment effects in the spatial water maze with both groups showed similar rates. B) Prenatally fruit-juice exposed rats did not locate the platform significantly faster than controls. C) Prenatally fruit-juice exposed rats crossed the platform location significantly more times than control rats. D) Proportion of rats that crossed the platform during the 30 sec probe task. As shown, significantly more animals crossed the platform location in the fruit-treated group. E) There was no difference in the amount of time spent in the target vs. non target locations of the maze when collapsed across groups nor were there any differences in this metric between the groups. * = *P*<0.05 fruit juice vs control.

In the modified spatial learning version of the water maze we employed, rats were required to learn and navigate to the hidden location of the submerged platform using the configuration of spatial cues in the room around the circular pool over 4 days, with 2 trials per day. The average latencies to swim to the platform location improved (i.e., decreased) across the four days of training, suggesting that spatial learning had taken place (Fig 3A). Indeed, collapsed across groups, there was a significant decrease in latencies across days and trials as analyzed using a 2-way repeated measures ANOVA (F_(7,560)_ = 10.0; *P*<0.0001). However, there were no significant differences in latency values between the offspring of fruit-fed dams and offspring of control dams (F_(1,80)_ = 0.26; *P* = 0.6), nor was there a significant interaction between treatments and trials (F_(7,560)_ = 0.88; *P* = 0.5). Similar results were obtained when analyzing data that were normalized by control data within cohorts. This shows that learning in this version of the circular water maze task appears to be unaffected by prenatal exposure to fruit juice.

### Visuo-spatial memory in circular water maze: Prenatal fruit juice treatment enhances memory

By analyzing behaviour in the probe trial where the platform was removed from the circular water maze we were able to extract several measures of spatial memory performance that included 1) the latency to cross the location at which the platform had been placed during learning trials, 2) the frequency of platform location crosses, 3) the total number of animals successfully crossing the platform location within the 30 second time probe test time frame, and 4) the time spent in the platform quadrant relative to other maze quadrants. While there was no discernable difference between treatment groups in the degree of learning in the circular water maze task, we found that prenatal exposure to fruit did appear to have some beneficial effects on spatial memory performance of offspring.

Rats prenatally exposed to fruit juice exhibited decreased latencies to the platform location as compared to control rats, though this effect was not significant (t_(80)_ = 1.97; *P* = 0.06; Fig 3B). Also consistent with marginally better memory performance in this group, we found that there was an overall significant increase of platform location crosses during the probe task, as compared to control rats (t_(80)_ = 3.37; *P* = 0.001; Fig 3C). When data were normalized to controls across cohorts, this effect was also apparent (t_(80)_ = 2.9, *P* = 0.004). More generally across control rats, there were fewer animals that crossed the platform location during the probe task compared to prenatally fruit-exposed rats (X_(1)_ = 16.2, *P*<0.0001, Fig 3D). While we were unable to find any significant improvements in terms of time spent in target quadrant vs time in other quadrants within the entire batch of animals (F_(1,160)_ = 0.68; *P* = 0.41; Fig 3E) this measure was likely contaminated by the large numbers of animals that did not show platform crosses in the first place. Perhaps not surprisingly, there was no effect of treatment (F_(1,160)_ = 0.001; *P* = 0.97) for this measure either.

To ensure that the increased number of platform crosses we observed were not due to a confound of the average swim speed of the rat, we analyzed the correlation between average swim speed of each animal (cm/s) and each of our probe trial measures: number of platform crosses, latency to the previous platform location and time in the platform quadrant. The only measure with a significant correlation with swim speed was latency (r = -0.21; *P* = 0.048), however, given that this relationship only accounted for 4% of the total variance, and that latency was not significantly different between fruit juice-exposed and control offspring, we do not consider swim speed to have biased our results. Moreover, when we normalized swim speed to cohort controls we found that there were no significant differences between prenatal fruit juice and control rats (t_(80)_ = 1.1, *P* = 0.24).

### Fear learning impaired in prenatal fruit juice rats compared to controls

**Fig 4:**
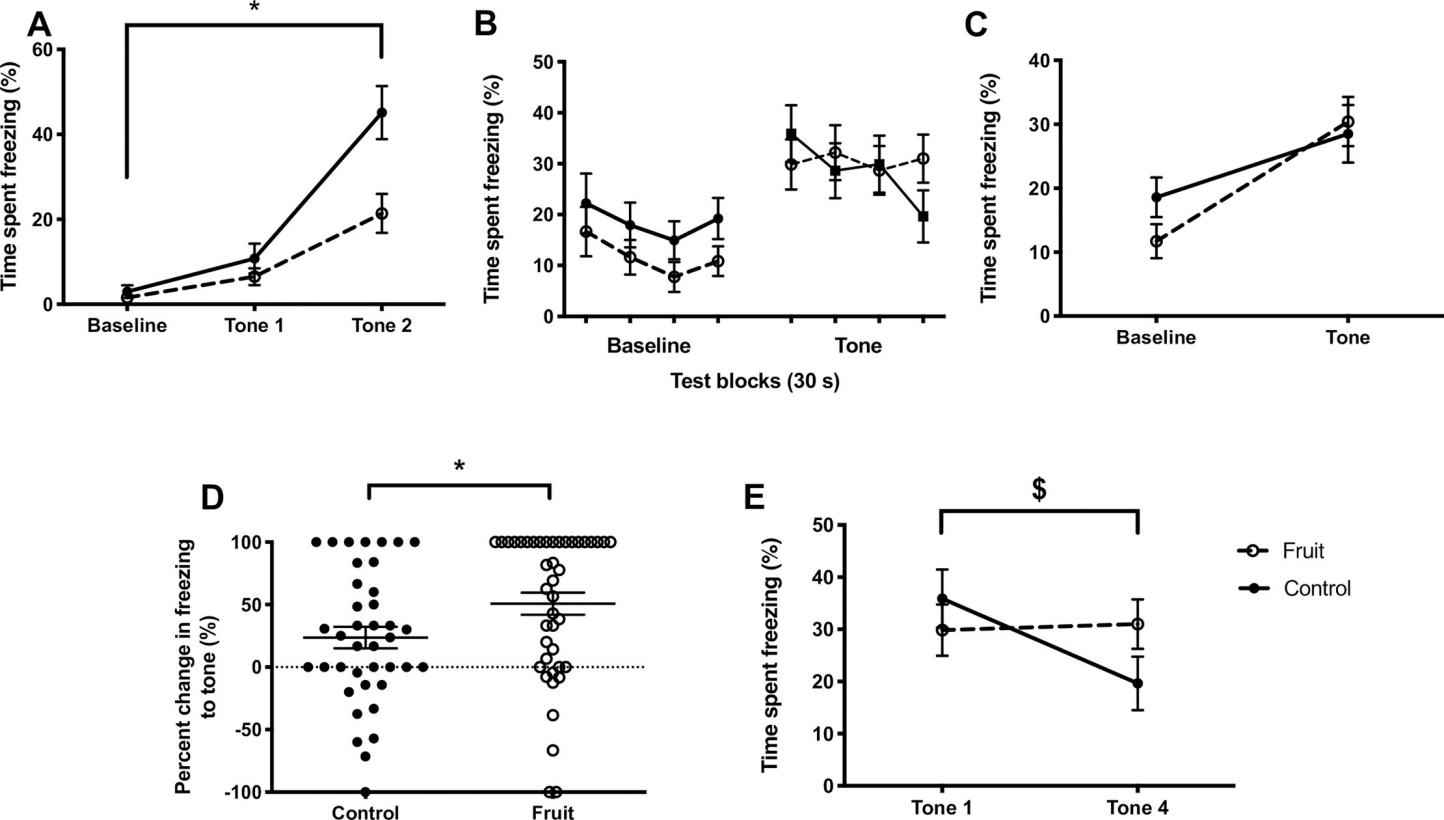
Fear conditioning: Measured by proportion of time spent freezing during UCS+CS pairings. A) During learning, all rats increased freezing with increased pairings of tone and shock. Overall, control rats showed stronger freezing during learning compared to rats exposed to prenatal fruit juice. B) Freezing during baseline and tone periods, broken down into 30 second blocks. During the memory task all rats increased freezing when hearing the tone previously associated with a foot shock. C) The average freezing across time periods for both baseline and cue presentation. D) Normalized freezing to cue presentation by factoring out baseline measures. Prenatally fruit-exposed rats showed increased freezing responses specifically to the tone, in comparison to the control rats. E) Freezing during the first and last 30 seconds of cue exposure showed that prenatally fruit-fed rats had greater consistency in fear responding to the cue, which had previously been co-terminated with a foot shock. * = *P*<0.05 fruit juice vs control. $ = interaction between treatment and time.

Fear-induced freezing was assessed during the presentation of the auditory cue during the conditioning procedure. As expected, during the conditioning trials themselves, all rats showed increases in freezing behaviour during the second repeated presentation of the conditioned stimulus (CS: tone) that had previously been paired with the unconditioned stimulus (UCS: foot shock) when compared to the baseline (pre-shock) period (F_(2,160)_ = 48.2; *P*<0.0001; Fig 4A). There was a significant effect of treatment (F_(1,80)_ = 8.8; *P* = 0.004), and significant interaction between time point and treatment (F_(2,160)_ = 6.3; *P* = 0.002) where control rats showed more freezing during the second exposure to the tone-shock pairing compared to prenatal fruit juice exposed rats. This suggests that control rats demonstrated stronger learning during the pairing procedure.

### Fear memory: Prenatal fruit juice exposed rats showed stronger cue-induced memory than control rats

Both groups of rats showed an increase in freezing in the alternate context (that was not associated with the UCS) after hearing the tone that was previously paired with the shock (F_(7,560)_ = 9.12; *P*<0.0001; Fig 4B). There were no overall significant differences in freezing measures between the groups across the full dataset, (F_(1,80)_ = 0.34; *P* = 0.55), and no significant interactions between the two groups across the data points measured (F_(7,560)_ = 1.6; *P* = 0.14) over time. These results were similar when the dataset was collapsed by averaging across both the baseline and cue presentation periods (Fig 4C). All rats showed an increase in freezing to the tone (F_(1,80)_ = 26.2; *P*<0.0001) but again there was no significant effect of treatment (F_(1,80)_ = 0.34; *P* = 0.55). Moreover, despite the tendency for the control rats to show more baseline freezing, there was no significant interaction between groups and condition (F_(1,80)_ = 2.5; *P* = 0.12).

Given the tendency for the control rats to show greater freezing in the baseline period, we further explored potential differences by normalizing data to this measure in order to specifically explore conditioned freezing to the conditioned stimulus (i.e., tone). When normalizing for overall freezing, the change in freezing to the tone was significantly increased in prenatal fruit juice exposed rats compared to controls (t_(80)_ = 2.2, *P* = 0.03, Fig 4D).

As rats varied their freezing behaviour across the full time frame of the tone presentation, we explored the consistency of freezing more explicitly. When comparing freezing to the tone during the first quarter of the tone period to the last quarter of the tone period there was a marginal, yet non-significant difference between the two timepoints (F_(1,80)_ = 3; *P* = 0.08), and a lack of effect across the groups (F_(1,80)_ = .21; *P* = 0.65). However, there was a significant interaction across time points and groups (F_(1,80)_ = 4.0; *P* = 0.04, 4E) suggesting that control rats showed a significant tendency to decrease their freezing with time with presentation of the auditory cue.

Altogether, these data suggest that despite weaker learning measures during the conditioning period, prenatal fruit-exposed rats demonstrated slightly stronger associative fear memory for the cue as measured by their normalized increase in cue responding, and a heightened consistency of freezing responses when re-exposed to the auditory stimulus in a different environment 24 hours following the original training.

### Gestational hydration influences on memory measures

Given the significant fluid intake differences across the fruit and control dams, we explored if hydration factors might have been involved in our reported cognitive differences. We correlated the net fluid intake of dams to the memory measures shown by their progeny in both spatial and fear conditioning tasks within both groups. We found no significant positive relationships in fluid intake to spatial (R^2^ = 0.001; *P* = 0.84 in control; R^2^ = 0.008; *P* = 0.56 in fruit juice) or emotional (R^2^ = 0.01; *P* = 0.51 in control; R^2^ = 0.0003; *P* = 0.90 in fruit juice) memory scores. Thus, it is unlikely that differences in fluid intake between fruit juice and control dams contributed to the prenatal fruit juice-induced behavior.

## Discussion

The offspring of pregnant rat dams exposed to a diet enriched with fruit (specifically orange and tomato-juice) during gestation exhibited moderately enhanced memory performance on both the circular water maze and cued fear memory tasks. Interestingly, we found no evidence for any advantage on learning in either of these tasks for this group. This suggests that prenatal fruit exposure confers some benefits in processes *unique* to memory (i.e, consolidation) and not in learning *per se*. Furthermore, these results follow and provide additional experimental validation of our prior study showing that a prenatal diet rich in lycopene and fructose correlates to enhanced cognitive outcomes in human progeny together with the experimental work showing enhanced memory in *Drosophila* [7].

While the effects initially observed in the CHILD study did not distinguish between whole fruit and juice intake when accounting for fruit consumption, a detailed multivariate analysis did not show any interactions with fiber and fruit intake on infant cognitive scores [7]. In the current study, we used only fruit juice in order to tractably quantify intake. It is clear that orange and tomato juice have differences in nutrient profiles compared to raw whole fruits [12]. For example, tomato juice contains increased iron, vitamin C, Thiamin, riboflavin, lycopene, and decreased fiber, lutein, and vitamin K compared to raw tomato fruit. Similarly, orange juice contains increased calcium, iron, phosphorus, Vitamin A, Vitamin E, arginine, proline, and decreased fiber, selenium, thiamin, beta carotene, cryptoxanthin, lutein, tryptophan, isoleucine, methionine, cysteine, tyrosine, histidine and glycine, as compared to raw orange fruit. However, these nutritional differences (notably in fiber, sugar, or lutein content), are likely not mediating the beneficial effects of prenatal fruit consumption in the CHILD study (reported in [7]), as we experimentally observed similar effects on cognition in both the *Drosophila* and rodent model using fruit juice as a supplement to the prenatal diet. Moreover, the previous research using *Drosophila* explicitly assessed sucrose supplementation alone and while this manipulation did enhance short-term performance in the olfactory conditioning task, it had no effect on long-term memory [7]. This suggests that in the prenatal fruit juice-treated rat offspring, memory (and to a lesser extent, learning) is insensitive to sucrose supplementation but likely more sensitive to the other nutrients present in the fruit juice. However, we did not explicitly test this in the present study. Future research using mammalian models should analyze the influence(s) of prenatal energy sources during pregnancy on cognitive neurodevelopment and brain metabolism in progeny.

The current study, having identified modest cognitive benefits in rats treated with prenatal fruit supplementation, thus prefaces future research identifying the “essential nutrients” responsible for the cognitive enhancements we have observed, as well as further characterization of the potential molecular underpinnings. Alimentary questionnaires from the CHILD study detailing the type of fruit most consumed, suggest that lycopene and fructose are important nutrients contributing to the beneficial effects observed in the current study. Surprisingly, the results from the Drosophila studies revealed that it was likely necessary that a combination of orange and tomato be present for the memory enhancement to be observed [7]. Therefore, it is possible that not only the combination of nutrients for a given fruit alone, but the combination of nutrients present in both orange and tomato juice work synergistically as an “entourage effect” [13]. For example, tomato juice has increased manganese, selenium, Vitamin C, thiamin, riboflavin, niacin, beta carotene, lycopene, lutein, threonine, isoleucine, methionine, cysteine, phenylalanine, tyrosine, histidine, and glutamic acid; and decreased sugar, calcium, Vitamin A, Vitamin E, monounsaturated fatty acids, arginine, and proline, compared to orange juice. Thus, it is possible that the nutrients in tomato juice confer benefits when boosted in combination with nutrients from orange juice, potentially enhancing bioavailability of nutrients [13] or synergistic actions of nutrients.

Both tomato juice (pH ~4.1), and orange juice (pH ~3.3) are acidic. The increased acidity of the juices may make many of the nutrients from either the juice or the rodent chow ingested more readily available to the blood and therefore the developing fetus [14]. However, the acidity of the juice may also cause heartburn in the pregnant dams, altering their behaviour. We did not assess for discomfort in dams caused by potential acid reflux due to juice ingestion, and it is unclear if the volumes of juice consumed would have induced acid reflux. Nevertheless, in pregnancy acid reflux is more common and exacerbated by acidic juices [15]. Therefore, we cannot rule out a potential confound of increased acid reflux during gestation in fruit juice-fed dams. However, we noted no evidence for conditioned taste aversions in consumption that would be expected in rats following ingestion of a discomforting novel taste, as fruit juice–fed rats drank stable, if not increasing volumes of juice throughout the pregnancy. Importantly, and perhaps related, diet also has an effect on the maternal gut microbiome which in turn has been shown to affect the pup microbiome and the cognitive outcome of offspring [16, 17]. In direct relation, tomato juice has been shown to have probiotic effects [18].

Given that the pregnant rats exposed to fruit juice drank more fluid than the control rats, we also cannot conclusively rule out the effects of gestational hydration differences across both conditions. Hydration has been shown to be an important factor for developing pups *in utero* [19]. However, we saw no relationship when correlating fluid intake of pregnant dams to the cognitive measures in the offspring, within each treatment group. Therefore, any possible fluctuations in gestational hydration as observed here appear to have had little influence on future cognitive abilities in offspring.

A major limitation in the current study is that only male rats were tested; and therefore, it is unclear if effects of prenatal fruit juice exposure extend to both sexes in rats, or may have differential effects across sexes. However, both the CHILD study and Drosophila study included both sexes, and did not detect prepubertal sex-differences for effects of prenatal exposure fruit [7], suggesting that at least in human and fruit fly models, prenatal fruit effects on cognition indeed generalize across both sexes. Future studies in rodents should attempt to characterize this influence in both sexes.

As others have reported differences in maternal care contributing to cognitive development of offspring [20, 21, 22, 23], we cannot rule out that changes in prenatal diet had behavioural effects in dams related to the post-natal care of offspring. We ensured that our dams were taken off fruit juice supplementation following delivery of the litter, and subsequently no active manipulation was taking place post-parturition. However, since maternal post-natal behaviours were not assessed in the current study we cannot absolutely exclude maternal care as a potential cause of the differences reported. Future experiments will be required to assess for the role of changes in maternal behavior (for example, activity level, sleep-wake cycle) during pregnancy, or after the delivery (frequency and type of interaction with pups).

Cognitive behavioral enhancement in wild-type animals is not common. We were therefore surprised that a simple prenatal dietary supplement of fruit juice would have any effects on cognitive behaviors in offspring. Nonetheless, these results recapitulate what was observed in the CHILD cohort study and after the active intervention in Drosophila. Therefore, we interpret this effect of prenatal exposure to fruit juice having as having long lasting implications for post-weaning cognitive measures, as their last exposure to fruit juice was *in utero* (18+ days earlier). Similarly, in the *Drosophila* model, prenatal fruit juice-exposed flies showed enhanced cognition, at 4 days old, which in a fruit fly is pre-adulthood, and pre-sexual maturity, however no assessments were conducted past this developmental time point. The CHILD study continues longitudinally, and recently found that 2-year-old infants showed beneficial effects of prenatal fruit on language development [24]. The authors of the most recent CHILD study publication hypothesized that such an effect was the result of persistent changes in neuronal circuits or molecular signaling that may be created by supplementing the prenatal fruit diet with fruit.

It should also be noted that the cognitive tasks used in the current study (the spatial version of the circular water maze and the fear conditioning task) assess functioning of different memory systems in mammals. The water maze is an assessment of spatial navigational memory, dependent on hippocampal functioning [25, 11], and the auditory-cued fear conditioning task is an emotional memory task, dependent on amygdala functioning [26]. Thus, expanding on the findings from [7], we show that prenatal fruit juice exposure has effects on mammalian memory systems, and thus likely a host of other behaviours that were not tested in our studies. Future studies would be well advised to use a battery of sensory, cognitive, social, and emotional responding tests to establish other behavioral effects of prenatal fruit juice.

We interpret that the moderate enhancement we observed in both spatial and fear memory (but not of learning) following prenatal fruit juice supplementation is due to an augmentation of pathways involved in consolidation mediated by the increased prenatal fruit intake. In the *Drosophila* model, prenatal fruit juice effects on cognition were tested in flies with mutations in the cAMP and PKA activation pathways, and the beneficial effects observed in the fruit condition were attenuated in these mutants [7]. This pathway is known to be highly conserved across species and similarly important for learning and memory [27], suggesting this may be the pathway promoted by the pre-natal fruit treatments in both fruit flies and rat. Another possibility, perhaps in concert with changes in molecular signaling processes, is that post-learning activity patterns that are known to play a role in memory consolidation may also be affected by pre-natal fruit exposure. Brain activity following a learning event is well-known to modulate the subsequent consolidation of this information, such that if post-learning activity is disrupted or boosted, memory expression will similarly show impairments or improvements, respectively [28, 29, 30]. Future studies testing this hypothesis could record from rats following learning to test for differences in brain activity or behaviours that may occur in the hours following the learning event. Altered neural activity following learning could be due to differences in behaviour following the learning event such as increased sleep, or playing behaviours [29, 30, 28], or due to physiological differences that may modulate neural activity following learning event (see [7]). Taken together these data suggest that prenatal fruit juice exposure may confer enhanced memory consolidation processes by altering intracellular mechanisms important for activity dependent synaptic plasticity [31], and cellular and molecular changes following a learning event.

Translating these results back to humans, the effects of prenatal diet on cognition over the human life span remain unclear. A recent meta-analysis examined effects of prenatal healthy diet on offspring cognitive outcomes up to 9 years, and only modest benefits of healthy gestational diet were maintained, with comparable effects between early infancy up to 9 years [5]. Indeed, infant verbal IQ scores at 12 months only modestly predict adult IQ [32]. However, it is currently unclear if early life intelligence modulated by prenatal fruit would be sustained as enhanced intelligence across the lifespan, or if other environmental factors that the child is exposed to across the lifespan, for example their own diet, would start to play a more prominent role in cognition as the child develops. For example, increased fruit and vegetable consumption across the lifespan has been linked to cognitive reserve and reduced cognitive impairment in the elderly [33, 34].

The confirmation of some of the previously reported benefits of prenatal fruit juice exposure in an experimental, mammalian rat model is of value for future studies assessing the precise neurodevelopmental period during pregnancy/gestation (and perhaps even the perinatal period) during which fruit juice-mediated effects on cognition are manifested. Rats have similar gestational stages to humans that can be divided into early, middle, and late phases [35]. However, rat parturition happens on the comparative timeline of the beginning of third trimester in humans. Therefore, in translating our results to humans, we suggest that the fruit mediated effects reported herein are related to gestational fruit consumption throughout the first and second trimester in human gestation, and further studies of fruit effects on late pregnancy in humans may be better modeled with post parturition fruit juice supplementation to rat dams. Future experiments should aim to determine which specific stage of brain development is important for the benefits of prenatal fruit juice observed here. In addition, the rodent model described here would be ideal for testing the effects of prenatal fruit juice in other models of neurodevelopmental disorders such as autism, intellectual disabilities, and epileptic encephalopathies, which impact cognitive development, in hopes of finding a developmental window in which prenatal dietary interventions could improve infant brain health and development, and therefore future cognitive or behavioral outcomes.
